# Trends in liver failure-related mortality among middle-aged and older adults with digestive system malignant tumors in the United States, 1999–2023

**DOI:** 10.3389/fonc.2026.1825036

**Published:** 2026-07-13

**Authors:** Ling Zhao, Jiawei Li, Zhengjun Cheng, Ao Ren

**Affiliations:** 1 Department of General Surgery, The Second People’s Hospital of Jiulongpo District, Chongqing, China; 2 Department of Hepatobiliary Surgery, The First Affiliated Hospital of Chongqing Medical University, Chongqing, China

**Keywords:** digestive system malignant tumors, joinpoint regression, liver failure, mortality trends, multiple cause-of-death analysis, population-based study

## Abstract

**Background:**

Digestive system malignant tumors (DSMTs) represent a major contributor to global cancer-related morbidity and mortality. With improving cancer survival, coexisting conditions and complications, including liver failure, have become increasingly relevant in mortality patterns among patients with DSMTs. However, population-level evidence on temporal trends and disparities in liver failure-related mortality remains limited.

**Methods:**

This population-based retrospective repeated cross-sectional study used data from the Centers for Disease Control and Prevention Wide-ranging Online Data for Epidemiologic Research (CDC WONDER) database. Death records from 1999 to 2023 were analyzed among patients aged ≥45 years with DSMTs, defined by ICD-10 codes C15-C26. Liver failure was identified using ICD-10 code K72 under a multiple cause-of-death framework. Age-adjusted mortality rates (AAMRs) were calculated using the 2000 U.S. standard population. Temporal trends were assessed using Joinpoint regression to estimate annual percent change (APC) and average annual percent change (AAPC). Analyses were stratified by sex, age group, race and ethnicity, urbanization level, Census region, and Health and Human Services (HHS) region.

**Results:**

From 1999 to 2023, a total of 82,796 liver failure-related deaths occurred among patients aged ≥45 years with DSMTs in the United States. Annual deaths declined from 4,131 in 1999 to 3,250 in 2023 (−21.33%). The AAMR decreased from 4.34 per 100,000 population (95% CI: 4.21–4.47) to 2.08 per 100,000 (95% CI: 2.01–2.15), corresponding to a significant overall decline (AAPC = −2.94%, 95% CI: −3.18 to −2.69). Although mortality declined across most subgroups, substantial heterogeneity persisted. Males consistently exhibited higher mortality than females, deaths were concentrated among older age groups, and liver failure-related deaths increased markedly among Hispanic individuals. Geographic analyses revealed pronounced regional variation, with attenuated declines or increasing trends in selected Census and HHS regions.

**Conclusions:**

Liver failure-related mortality among middle-aged and older patients with DSMTs in the United States has declined substantially over the past two decades. However, this improvement has slowed in recent years and remains uneven across populations and regions. These findings support continued attention to liver function monitoring, liver disease risk assessment, and targeted surveillance in high-risk populations and regions.

## Introduction

Digestive system malignant tumors (DSMTs) refer to malignant neoplasms arising from organs of the digestive system, including the esophagus, stomach, small intestine, colon, rectum, anal canal, liver, extrahepatic biliary tract, and pancreas (1–3). According to GLOBOCAN 2022 estimates, DSMTs account for approximately one-quarter of newly diagnosed cancer cases worldwide while contributing to more than one-third of all cancer-related deaths (4). Major subtypes such as colorectal, gastric, esophageal, liver, and pancreatic cancers collectively constitute a substantial global disease burden and place sustained pressure on healthcare systems and public health resources (5, 6).

With progressive population aging and the persistent influence of lifestyle-related and metabolism-related risk factors, the mortality burden of DSMTs remains considerable among middle-aged and older patients (7, 8). Although advances in cancer screening and multimodal treatment have contributed to declining mortality rates for certain DSMTs, these improvements are unevenly distributed across demographic and geographic strata. As a result, mortality patterns associated with DSMTs have become increasingly complex (9–11). For patients with resectable disease, surgical resection remains an important potentially curative treatment modality, while chemotherapy, radiotherapy, targeted therapy, immunotherapy, and supportive care are used according to tumor type, disease stage, prior treatment, performance status, and surgical candidacy. Despite these advances, many DSMTs continue to be diagnosed at advanced stages, and coexisting conditions and complications recorded at death have become increasingly relevant, particularly among older patients (12, 13).

Liver failure is a severe clinical syndrome characterized by acute or progressive hepatic decompensation and is not uncommon among patients with DSMTs (14–16). In patients with DSMTs, liver failure may be recorded in several clinical contexts, including primary hepatic malignancy, hepatic metastatic burden, treatment-related hepatotoxicity, preexisting chronic liver disease, and metabolic disorders. Anatomically, the liver’s position within the portal venous system makes it a common site of metastatic involvement for several DSMTs, and extensive hepatic involvement may contribute to progressive hepatic decompensation (17, 18). In addition, liver injury associated with chemotherapy, targeted therapy, and immunotherapy has become increasingly relevant as systemic therapies have expanded (19–21). Older patients with DSMTs may also have underlying chronic liver disease or metabolic disorders, further increasing vulnerability to hepatic decompensation (22, 23). Once liver failure occurs, it can substantially limit the feasibility of subsequent antitumor therapies and significantly increase both short-term and long-term mortality risks. However, population-level evidence regarding liver failure-related mortality and its temporal trends among individuals with DSMTs remains scarce.

Therefore, using the Centers for Disease Control and Prevention Wide-ranging Online Data for Epidemiologic Research (CDC WONDER) database and a multiple cause-of-death analytical framework, this study was designed as a descriptive mortality surveillance analysis. Our objective was to quantify national temporal trends and disparities in deaths in which both DSMTs and liver failure were recorded on death certificates among middle-aged and older adults in the United States. Given improvements in cancer screening, multimodal treatment, perioperative care, and supportive management, we hypothesized that liver failure-related mortality may have declined over time, but that the magnitude of decline would vary across demographic and geographic subgroups. By identifying population groups and regions with persistent or unfavorable mortality patterns, this study may help inform future tumor site-specific research, liver function surveillance strategies, and targeted public health interventions.

## Methods

### Study population

This population-based retrospective repeated cross-sectional study utilized data from the publicly available CDC WONDER database. The database is compiled by the National Center for Health Statistics (NCHS) based on nationwide death certificate records, covering all 50 U.S. states and the District of Columbia. Death records registered between 1999 and 2023 were included. The study population was restricted to patients aged ≥45 years at death with DSMTs, defined by ICD-10 codes. Because CDC WONDER provides fully de-identified public health surveillance data, this study was exempt from institutional review board approval and informed consent requirements.

### Data extraction

Mortality data were extracted using the multiple cause-of-death module within the CDC WONDER database. DSMTs were identified using ICD-10 codes C15-C26 (24), and liver failure was defined using ICD-10 code K72 (25). Deaths in which both DSMTs and liver failure were listed anywhere on the death certificate, either as the underlying cause of death or as contributing causes of death, were classified as liver failure-related deaths (26, 27). This multiple cause-of-death definition identifies deaths in which DSMTs and liver failure were co-recorded on the death certificate and was used to quantify population-level mortality patterns rather than to infer a causal relationship between DSMTs and liver failure.

Data were stratified by age, sex, race and ethnicity, urbanization level, Census region, and Health and Human Services (HHS) region. Extracted variables included year of death, death counts, crude mortality rates with 95% confidence intervals (CIs), and age-adjusted mortality rates (AAMRs) with 95% CIs. Age stratification was conducted using ten-year age groups as defined by CDC WONDER. Race and ethnicity were classified according to CDC WONDER standard categories, including Hispanic/Latino, non-Hispanic White, non-Hispanic Black, and non-Hispanic Other (28, 29). Urbanization levels were defined using the 2013 National Center for Health Statistics (NCHS) urban-rural classification scheme, with Large Central Metro, Large Fringe Metro, Medium Metro, and Small Metro classified as urban areas, and Micropolitan (nonmetro) and Noncore (nonmetro) classified as nonurban areas (30, 31).

### Statistical analysis

AAMRs were calculated using direct standardization to the 2000 U.S. standard population. In age-stratified analyses, because CDC WONDER does not support calculation of AAMRs for specific age groups, crude mortality rates were used to describe temporal trends (32). Due to the unavailability of urbanization-stratified AAMR data for the years 2021-2023, trend analyses by urbanization level were restricted to data from 1999 through 2020 (33). Percent change in deaths was calculated using the following formula: [(deaths in 2023 – deaths in 1999)/deaths in 1999] × 100%. Temporal trends were assessed using Joinpoint regression software (National Cancer Institute) to estimate annual percent changes (APCs), average annual percent changes (AAPCs), and 95% CIs. Statistical significance was defined as p < 0.05. All analyses were conducted using R software (Version 4.5.2).

## Results

### Overall trends

From 1999 to 2023, a total of 82,796 liver failure-related deaths were identified among patients aged ≥45 years with DSMTs in the United States. The annual number of deaths declined from 4,131 in 1999 to 3,250 in 2023, representing an overall decrease of 21.33% (**Table 1**). Although year-to-year fluctuations were observed, the long-term pattern demonstrated a sustained reduction in the population-level burden of deaths involving both DSMTs and liver failure.

**Table 1:** Liver failure-related deaths and age-adjusted mortality rates among patients with digestive system malignant tumors in the United States.

Characteristic	Deaths			AAMR		
	1999	2023	Percent change (%)	1999 (95% CI)	2023 (95% CI)	AAPC (95% CI)
Overall	4131	3250	-21.33	4.34 (4.21 to 4.47)	2.08 (2.01 to 2.15)	-2.94 (-3.18 to -2.69)*
Sex						
Female	1608	1118	-30.47	2.94 (2.80 to 3.09)	1.40 (1.31 to 1.48)	-3.05 (-3.43 to -2.68)*
Male	2523	2132	-15.50	6.06 (5.82 to 6.30)	2.92 (2.79 to 3.05)	-2.90 (-3.21 to -2.60)*
Age^1^						
45–54 years	578	357	-38.24	1.58 (1.45 to 1.71)	0.88 (0.79 to 0.97)	-2.49 (-3.92 to -1.03)*
55–64 years	871	941	8.04	3.66 (3.42 to 3.91)	2.25 (2.10 to 2.39)	-2.07 (-2.87 to -1.26)*
65–74 years	1297	1161	-10.49	7.04 (6.66 to 7.42)	3.35 (3.15 to 3.54)	-2.89 (-3.54 to -2.24)*
75–84 years	1048	611	-41.70	8.57 (8.05 to 9.09)	3.33 (3.06 to 3.59)	-3.43 (-3.99 to -2.87)*
85+ years	337	180	-46.59	8.11 (7.25 to 8.98)	2.91 (2.48 to 3.33)	-3.93 (-5.03 to -2.81)*
Race						
Hispanic	302	463	53.31	5.77 (5.10 to 6.45)	2.54 (2.30 to 2.77)	-3.11 (-3.90 to -2.32)*
NH Black	456	432	-5.26	5.34 (4.85 to 5.84)	2.55 (2.30 to 2.79)	-2.88 (-3.35 to -2.40)*
NH White	3141	2116	-32.63	3.98 (3.84 to 4.12)	1.97 (1.89 to 2.06)	-2.92 (-3.21 to -2.62)*
NH Other	222	234	5.41	7.48 (6.45 to 8.51)	2.15 (1.87 to 2.42)	-4.84 (-5.80 to -3.87)*
Urbanization^2^						
Metropolitan	3429	2730	-20.38	4.42 (4.27 to 4.57)	2.01 (1.93 to 2.09)	-3.45 (-3.76 to -3.13)*
Nonmetropolitan	702	520	-25.93	3.95 (3.66 to 4.25)	2.37 (2.17 to 2.57)	-2.00 (-2.55 to -1.45)*
Census region						
Northeast	884	557	-36.99	4.48 (4.18 to 4.77)	1.99 (1.82 to 2.16)	-3.05 (-3.56 to -2.54)*
Midwest	811	638	-21.33	3.63 (3.38 to 3.88)	2.01 (1.85 to 2.17)	-2.35 (-2.87 to -1.84)*
South	1419	1188	-16.28	4.20 (3.98 to 4.42)	2.00 (1.89 to 2.12)	-2.88 (-3.31 to -2.46)*
West	1017	867	-14.75	5.23 (4.91 to 5.55)	2.48 (2.31 to 2.65)	-3.33 (-3.76 to -2.89)*
HHS region						
HHS1	280	149	-46.79	5.50 (4.85 to 6.14)	1.95 (1.63 to 2.27)	-3.56 (-5.12 to -1.97)*
HHS2	400	251	-37.25	4.11 (3.71 to 4.52)	1.82 (1.59 to 2.04)	-3.15 (-3.74 to -2.55)*
HHS3	447	336	-24.83	4.41 (4.00 to 4.82)	2.25 (2.00 to 2.49)	-2.90 (-3.59 to -2.21)*
HHS4	714	629	-11.90	3.72 (3.45 to 4.00)	1.85 (1.71 to 2.00)	-2.85 (-3.26 to -2.43)*
HHS5	602	469	-22.09	3.49 (3.21 to 3.77)	1.88 (1.70 to 2.05)	-2.56 (-3.12 to -2.01)*
HHS6	484	406	-16.12	4.75 (4.33 to 5.18)	2.26 (2.03 to 2.48)	-2.45 (-3.35 to -1.54)*
HHS7	185	147	-20.54	3.91 (3.34 to 4.47)	2.20 (1.84 to 2.57)	-2.03 (-2.90 to -1.15)*
HHS8	106	138	30.19	3.84 (3.11 to 4.57)	2.64 (2.19 to 3.09)	-1.00 (-2.05 to 0.06)
HHS9	797	514	-35.51	6.24 (5.81 to 6.68)	2.24 (2.05 to 2.44)	-4.55 (-5.14 to -3.94)*
HHS10	116	211	81.90	3.18 (2.60 to 3.76)	3.10 (2.67 to 3.53)	0.03 (-0.42 to 0.48)

^1^ For age groups, AAMRs were replaced by crude mortality rates, and AAPCs were calculated based on crude mortality rates. ^2^For urbanization level, AAMR data for 2023 were unavailable; therefore, 2020 values were used, and AAPCs were calculated for 1999–2020. Asterisks (*) indicate statistical significance. AAMR, age-adjusted mortality rate; AAPC, average annual percent change; CI, confidence interval; HHS, U.S. Department of Health and Human Services; NH, non-Hispanic.

**Figure 1:**
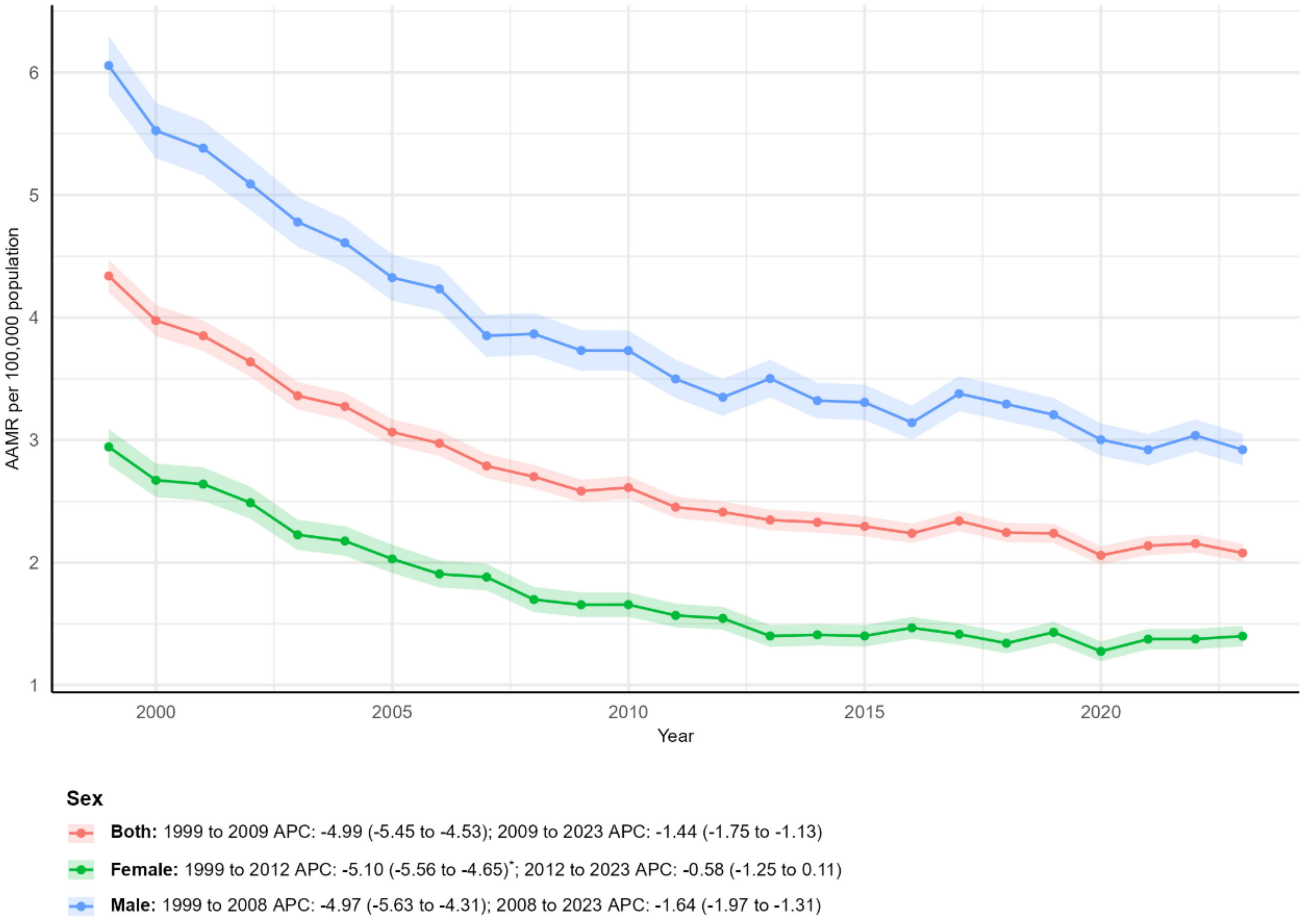
Trends in AAMR of liver failure-related deaths among patients with DSMTs in the United States from 1999 to 2023, stratified by sex. AAMR, age-adjusted mortality rate; APC, annual percent change; DSMTs, digestive system malignant tumors.

Consistent with changes in death counts, the AAMR decreased from 4.34 per 100,000 population (95% CI: 4.21–4.47) in 1999 to 2.08 per 100,000 population (95% CI: 2.01–2.15) in 2023 (**Table 1**). Joinpoint regression analysis revealed a statistically significant overall downward trend, with an AAPC of −2.94% (95% CI: −3.18 to −2.69; p < 0.001) (**Figure 1**). Segmented Joinpoint analysis further showed a two-phase pattern, with a steeper decline from 1999 to 2009 (APC = −4.99%) followed by a slower but persistent decline from 2009 to 2023 (APC = −1.44%). This attenuation after 2009 indicates that the reduction in liver failure-related mortality slowed over time rather than continuing at the earlier pace.

### Sex

Sex-stratified analyses showed that liver failure-related deaths were consistently higher among males than females throughout the study period. From 1999 to 2023, deaths among males declined from 2,523 to 2,132 (−15.50%), whereas deaths among females declined from 1,608 to 1,118 (−30.47%) (**Table 1**), indicating a more pronounced relative reduction among females.

Across all study years, males exhibited higher AAMRs than females. Among males, the AAMR declined from 6.06 per 100,000 (95% CI: 5.82–6.30) in 1999 to 2.92 per 100,000 (95% CI: 2.79–3.05) in 2023. Among females, the AAMR decreased from 2.94 per 100,000 (95% CI: 2.80–3.09) to 1.40 per 100,000 (95% CI: 1.31–1.48) (**Table 1**). Joinpoint regression confirmed significant overall declines for both sexes, with an AAPC of −2.90% (95% CI: −3.21 to −2.60; p < 0.001) for males and −3.05% (95% CI: −3.43 to −2.68; p < 0.001) for females (**Figure 1**). Similar attenuation of decline was observed in sex-stratified analyses. Among males, the AAMR declined steeply from 1999 to 2008 (APC = −4.97%), followed by a slower decline from 2008 to 2023 (APC = −1.64%). Among females, the AAMR declined markedly from 1999 to 2012 (APC = −5.10%), followed by a much slower later-period decline from 2012 to 2023 (APC = −0.58%).

### Age

Across age groups, liver failure-related deaths were predominantly concentrated among older patients with DSMTs. Over the entire study period, the highest cumulative number of deaths occurred in the 55–64 age group (24,198 deaths), followed by the 65–74 age group (24,081 deaths), whereas the lowest number was observed among patients aged ≥85 years (5,931 deaths). This lower cumulative death count should be interpreted in the context of the smaller population size in the ≥85 age group and does not necessarily indicate a lower mortality burden. From 1999 to 2023, the largest relative decline in deaths occurred in the ≥85 age group, decreasing from 337 to 180 deaths (−46.59%), followed by the 75–84 age group (from 1,048 to 611; −41.70%) and the 45–54 age group (from 578 to 357; −38.24%). In contrast, the 55–64 age group experienced a modest increase in deaths, from 871 to 941 (+8.04%) (**Table 1**).

**Figure 2:**
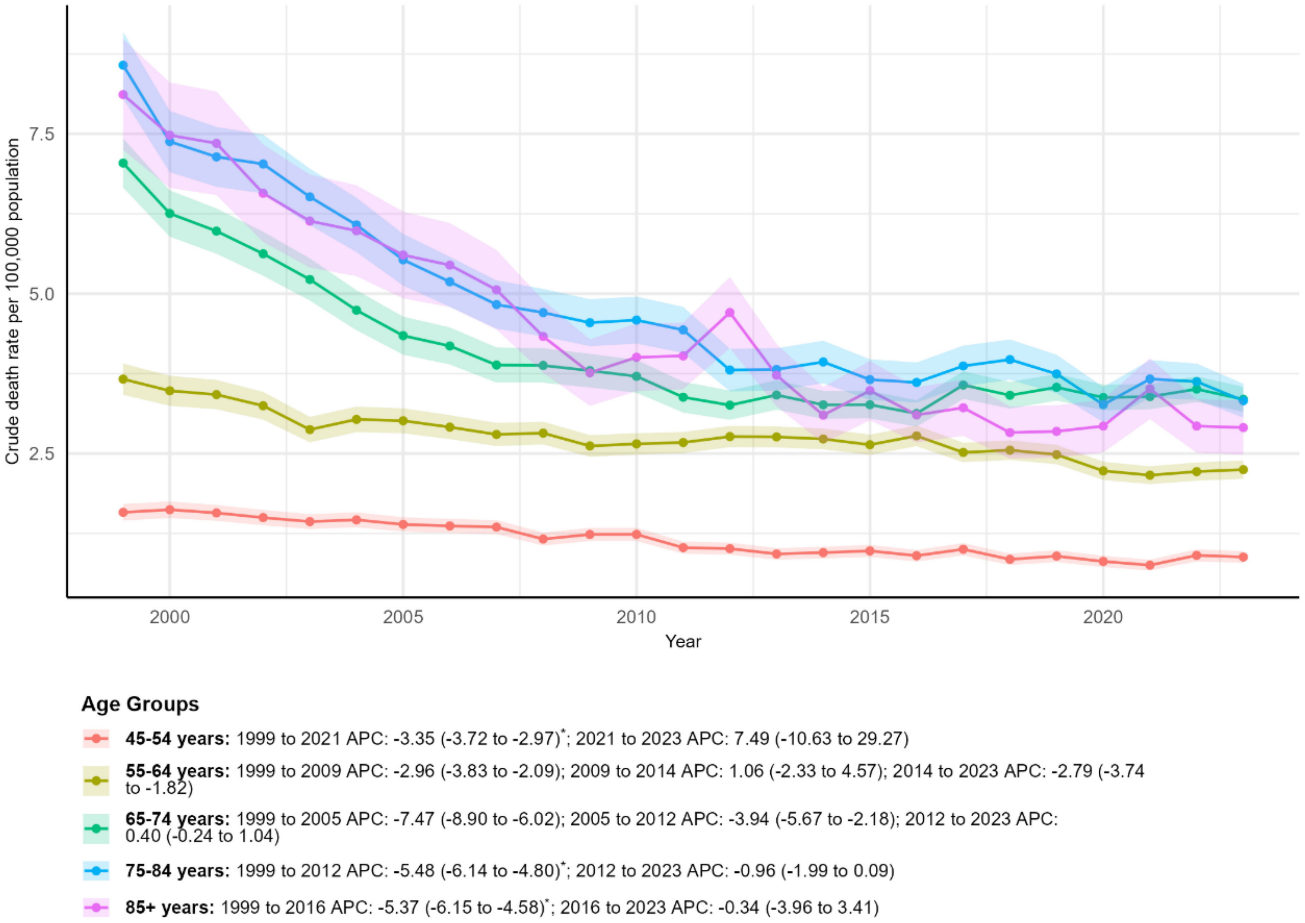
Trends in crude mortality rate of liver failure-related deaths among patients with DSMTs in the United States from 1999 to 2023, stratified by age group. AAMR, age-adjusted mortality rate; APC, annual percent change; DSMTs, digestive system malignant tumors.

The most pronounced decline was observed in the ≥85 age group, with crude mortality rates decreasing from 8.11 per 100,000 (95% CI: 7.25–8.98) in 1999 to 2.91 per 100,000 (95% CI: 2.48–3.33) in 2023, corresponding to the steepest overall decline (AAPC = −3.93%; 95% CI: −5.03 to −2.81; p < 0.001). Similarly, the 75–84 age group experienced a substantial reduction, with crude mortality rates declining from 8.57 (95% CI: 8.05–9.09) to 3.33 (95% CI: 3.06–3.59) per 100,000 (AAPC = −3.43%; 95% CI: −3.99 to −2.87; p < 0.001). In contrast, the smallest decline was observed in the 55–64 age group, where crude mortality rates decreased from 3.66 (95% CI: 3.42–3.91) to 2.25 (95% CI: 2.10–2.39) per 100,000 (AAPC = −2.07%; 95% CI: −2.87 to −1.26; p < 0.001) (**Table 1**). Joinpoint regression showed that age-specific mortality trends generally followed a pattern of early decline followed by attenuation or stabilization in later years (**Figure 2**). This pattern was particularly evident among patients aged ≥75 years, in whom steeper early reductions contributed to a gradual convergence of mortality rates across age strata. Accordingly, the range of crude mortality rates across age groups narrowed from 1.58–8.57 per 100,000 in 1999 to 0.88–3.35 per 100,000 in 2023.

### Race and ethnicity

Marked racial and ethnic disparities were observed in liver failure-related mortality among middle-aged and older patients with DSMTs. Over the study period, non-Hispanic White individuals accounted for the largest cumulative number of deaths (58,522). However, this larger absolute number of deaths should be interpreted in the context of population size and demographic composition, as non-Hispanic White individuals consistently demonstrated among the lowest AAMRs throughout the study period. From 1999 to 2023, deaths among non-Hispanic White individuals declined substantially from 3,141 to 2,116 (−32.63%), while deaths among non-Hispanic Black individuals declined modestly from 456 to 432 (−5.26%). In contrast, deaths among Hispanic individuals increased markedly, rising from 302 to 463 (+53.31%), and deaths among non-Hispanic Other groups increased slightly from 222 to 234 (+5.41%) (**Table 1**).

**Figure 3:**
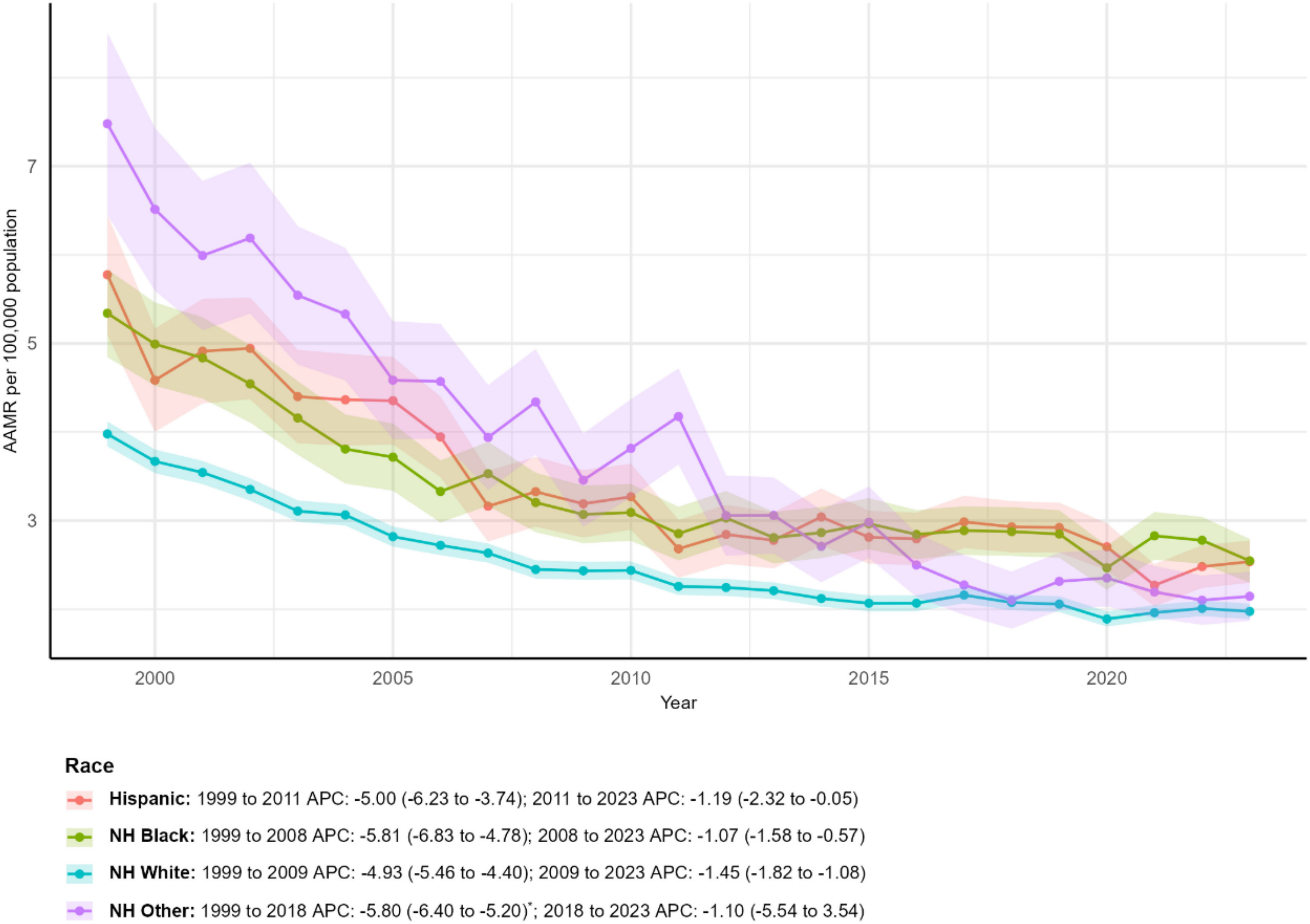
Trends in AAMR of liver failure-related deaths among patients with DSMTs in the United States from 1999 to 2023, stratified by race. AAMR, age-adjusted mortality rate; APC, annual percent change; DSMTs, digestive system malignant tumors.

Despite differences in death counts, AAMRs declined across all racial and ethnic groups during the study period. Among non-Hispanic White individuals, the AAMR decreased from 3.98 to 1.97 per 100,000 (AAPC = −2.92%; 95% CI: −3.21 to −2.62; p < 0.001). Comparable declines were observed among non-Hispanic Black individuals (from 5.34 to 2.55 per 100,000; AAPC = −2.88%, 95% CI: −3.35 to −2.40, p < 0.001), Hispanic individuals (from 5.77 to 2.54 per 100,000; AAPC = −3.11%, 95% CI: −3.90 to −2.32, p < 0.001), and non-Hispanic Other groups (from 7.48 to 2.15 per 100,000; AAPC = −4.84%, 95% CI: −5.80 to −3.87, p < 0.001) (**Table 1**). Joinpoint regression demonstrated steeper early declines followed by attenuated reductions across racial and ethnic groups (**Figure 3**). The non-Hispanic Other group showed the steepest long-term decline (AAPC = −4.84%) and shifted from having the highest AAMR in 1999 to among the lower mortality groups by 2023.

### Urbanization

Urbanization-stratified analyses showed that the majority of liver failure-related deaths occurred in metropolitan areas (59,883 deaths) over the study period. From 1999 to 2020, deaths declined in both metropolitan and nonmetropolitan areas, decreasing from 3,429 to 2,730 (−20.38%) in metropolitan areas and from 702 to 520 (−25.93%) in nonmetropolitan areas, indicating a slightly greater relative reduction in nonmetropolitan regions (**Table 1**).

**Figure 4:**
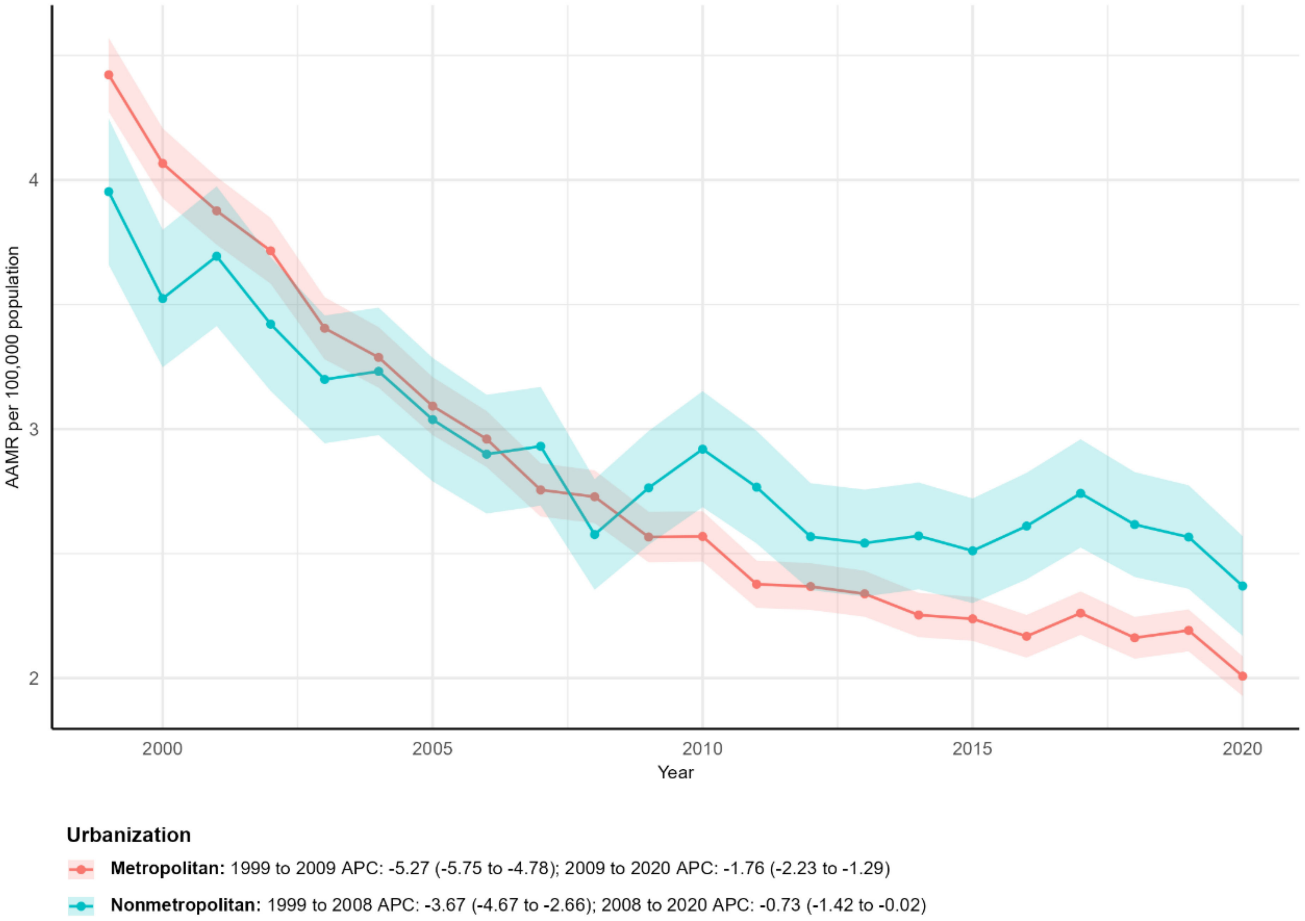
Trends in AAMR of liver failure-related deaths among patients with DSMTs in the United States from 1999 to 2020, stratified by urbanization level. AAMR, age-adjusted mortality rate; APC, annual percent change; DSMTs, digestive system malignant tumors.

Correspondingly, AAMRs declined significantly in both settings. In metropolitan areas, the AAMR decreased from 4.42 to 2.01 per 100,000 (AAPC = −3.45%; 95% CI: −3.76 to −3.13; p < 0.001), whereas in nonmetropolitan areas it declined from 3.95 to 2.37 per 100,000 (AAPC = −2.00%; 95% CI: −2.55 to −1.45; p < 0.001) (**Table 1**). Joinpoint regression revealed that both metropolitan and nonmetropolitan areas experienced early declines followed by attenuation in later years (**Figure 4**). In metropolitan areas, the AAMR declined rapidly from 1999 to 2009 (APC = −5.27%), followed by a slower decline from 2009 to 2020 (APC = −1.76%). In nonmetropolitan areas, the AAMR declined from 1999 to 2008 (APC = −3.67%), but the subsequent decline from 2008 to 2020 was much weaker (APC = −0.73%), suggesting a near-plateau pattern in the later period. These findings indicate that improvements slowed in both urbanization strata, with more pronounced deceleration in nonmetropolitan areas.

### Census region

Across Census regions, the South contributed the highest cumulative number of liver failure-related deaths (28,649), whereas the Northeast had the lowest (16,168). From 1999 to 2023, liver failure-related deaths declined in all four Census regions. The largest relative reduction was observed in the Northeast, where deaths decreased from 884 to 557 (−36.99%). Smaller declines were observed in the Midwest (−21.33%), South (−16.28%), and West (−14.75%) (**Table 1**).

**Figure 5:**
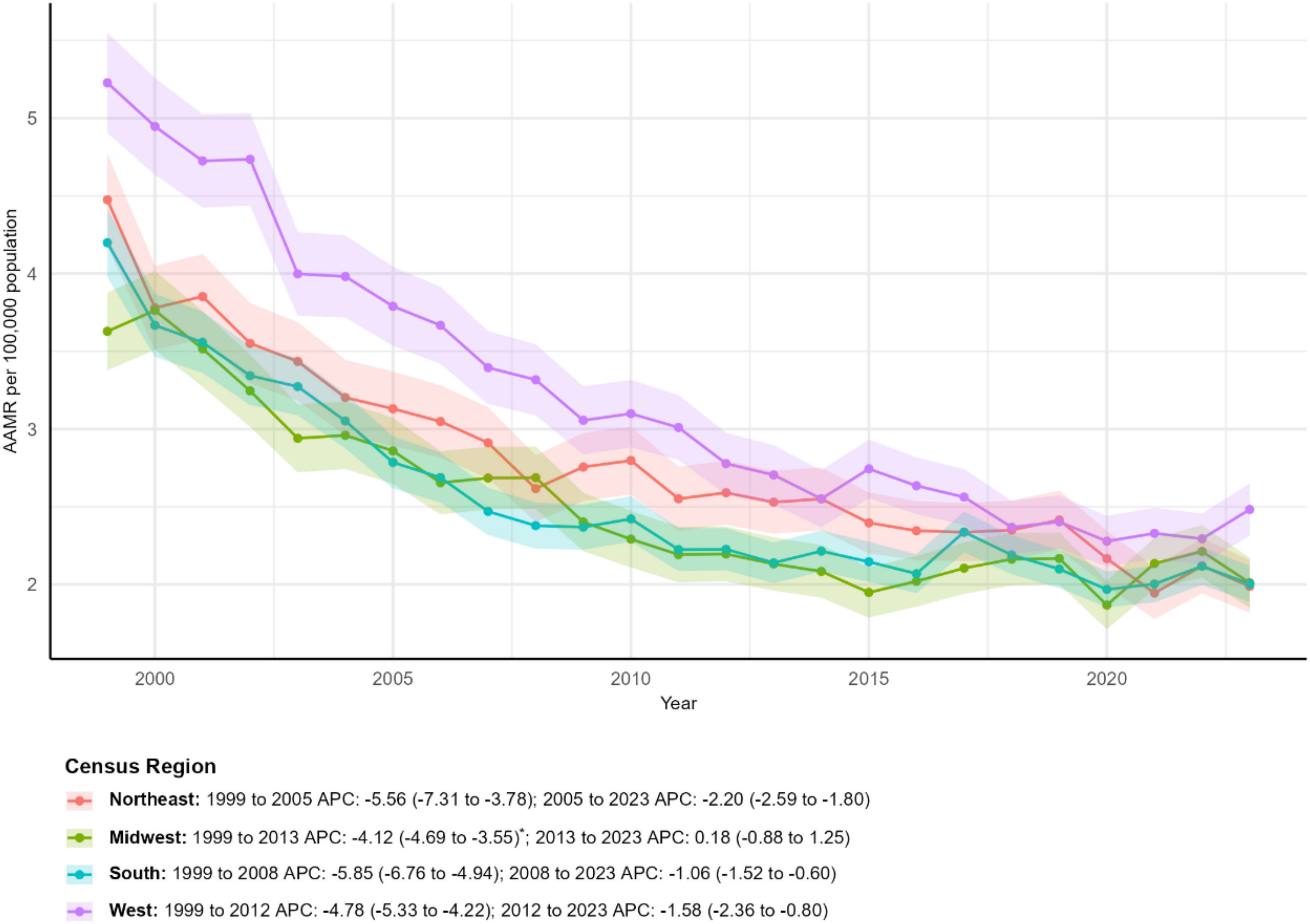
Trends in AAMR of liver failure-related deaths among patients with DSMTs in the United States from 1999 to 2023, stratified by census region. AAMR, age-adjusted mortality rate; APC, annual percent change; DSMTs, digestive system malignant tumors.

Consistent with these patterns, AAMRs declined significantly across all Census regions. The Northeast experienced a reduction from 4.48 to 1.99 per 100,000 (AAPC = −3.05%; 95% CI: −3.56 to −2.54; p < 0.001), while significant declines were also observed in the Midwest (AAPC = −2.35%; 95% CI: −2.87 to −1.84; p < 0.001), South (AAPC = −2.88%; 95% CI: −3.31 to −2.46; p < 0.001), and West (AAPC = −3.33%; 95% CI: −3.76 to −2.89; p < 0.001) (**Table 1**). Joinpoint regression showed that all Census regions experienced relatively steep early declines followed by slower subsequent trends, although the timing of attenuation varied across regions (**Figure 5**). The Northeast declined rapidly from 1999 to 2005 (APC = −5.56%) and continued to decrease from 2005 to 2023 at a more moderate pace (APC = −2.20%), indicating a relatively sustained downward trajectory. The Midwest showed a marked decline from 1999 to 2013 (APC = −4.12%), followed by a near-stable pattern from 2013 to 2023. The South declined steeply from 1999 to 2008 (APC = −5.85%) and then more slowly from 2008 to 2023 (APC = −1.06%). The West also showed a pronounced decline from 1999 to 2012 (APC = −4.78%), followed by a slower decline from 2012 to 2023 (APC = −1.58%); however, it generally maintained among the highest AAMRs across regions throughout much of the study period.

### HHS region

**Figure 6:**
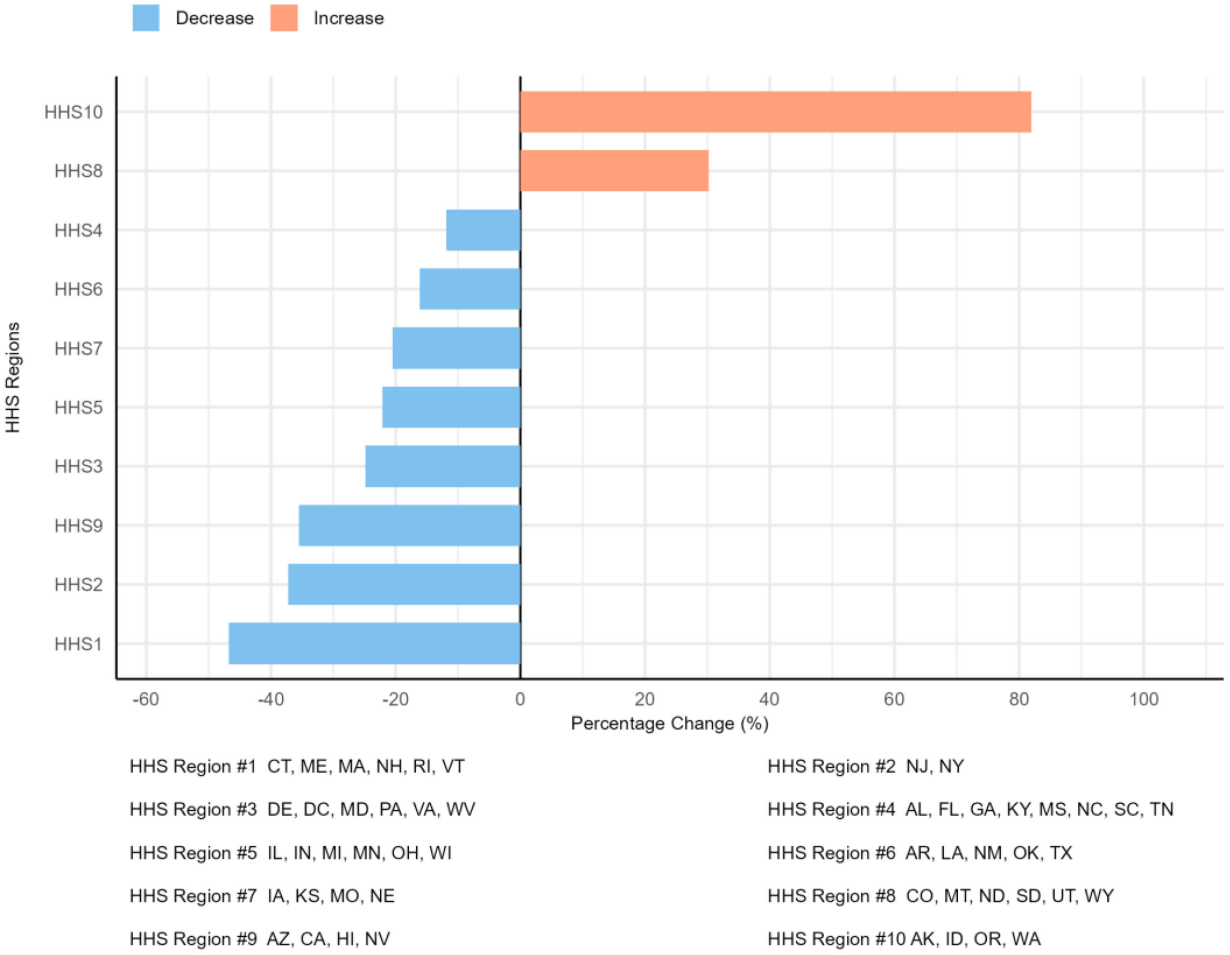
Percent change in liver failure-related deaths among patients with DSMTs in the United States from 1999 to 2023, stratified by HHS region. Full state names for each HHS region are provided in **Supplementary Table 1**. DSMTs, digestive system malignant tumors.

Substantial geographic heterogeneity was observed across HHS regions. From 1999 to 2023, most HHS regions experienced declines in liver failure-related deaths. The largest relative decrease occurred in HHS Region 1, where deaths declined from 280 to 149 (−46.79%), followed by HHS Region 2, with a reduction from 400 to 251 deaths (−37.25%). In contrast, deaths increased in HHS Region 8 from 106 to 138 (+30.19%) and more markedly in HHS Region 10 from 116 to 211 (+81.90%), indicating emerging regional disparities in mortality burden (**Figure 6**).

AAMRs generally declined across HHS regions, although the magnitude and statistical significance of these trends varied. The steepest long-term decline was observed in HHS Region 9 (AAPC = −4.55%; 95% CI: −5.14 to −3.94; p < 0.001), followed by HHS Region 1 (AAPC = −3.56%; 95% CI: −5.12 to −1.97; p < 0.001) and HHS Region 2 (AAPC = −3.15%; 95% CI: −3.74 to −2.55; p < 0.001). In contrast, HHS Region 8 showed a modest but statistically non-significant decline (AAPC = −1.00%; 95% CI: −2.05 to 0.06; p = 0.065), while HHS Region 10 showed a largely stable trend with no significant long-term change in mortality (AAPC = 0.03%; 95% CI: −0.42 to 0.48; p = 0.895) (**Table 1**).

## Discussion

This population-based study systematically evaluated temporal trends and disparities in liver failure-related mortality among U.S. adults aged ≥45 years with DSMTs from 1999 to 2023, using a multiple cause-of-death analytical framework based on national mortality data from CDC WONDER. Within the pooled DSMT category, both the number of deaths involving liver failure and liver failure-related mortality rates declined substantially during the study period. However, these findings should be interpreted as pooled mortality surveillance patterns rather than evidence of uniform declines across individual digestive tumor sites. The improvements were uneven across population subgroups and geographic regions, with varying degrees of trend deceleration, stabilization, or localized increases observed in recent years. These findings indicate that, despite advances in the comprehensive management of DSMTs, liver failure remains an important non-tumor-specific condition in mortality data and continues to impose a meaningful mortality burden in selected populations and regions.

The observed decline in liver failure-related mortality is likely multifactorial. From a population-level perspective, the widespread implementation of screening programs for DSMTs has improved early detection, enabling some patients to avoid prolonged high tumor burden or extensive hepatic involvement that can progressively compromise liver function (34, 35). In addition, advances in perioperative care and multidisciplinary management have facilitated earlier recognition and intervention for hepatic dysfunction (36, 37). Because these pathways may coexist and cannot be distinguished in death certificate data, the observed trends should be interpreted as pooled population-level mortality surveillance patterns rather than evidence of a shared biological mechanism across all DSMTs. Together, these factors may partly explain the overall decline observed in this study. Notably, Joinpoint regression revealed that mortality declined most rapidly during the early study period, followed by a marked slowing in more recent years. This temporal pattern suggests that earlier improvements may have achieved substantial initial gains, whereas further reductions may require more targeted strategies for patients and regions with persistent or unfavorable mortality patterns.

Substantial heterogeneity was observed across population subgroups, particularly by sex, age, and race/ethnicity, highlighting differences in underlying risk profiles and access to healthcare. Males consistently experienced higher liver failure-related mortality than females, potentially reflecting sex-related differences in underlying liver disease and metabolic risk profiles (38, 39). Because CDC WONDER does not provide individual-level data on tumor-site distribution, liver disease etiology, treatment exposure, or baseline liver function, this sex disparity should be interpreted as a descriptive mortality pattern rather than evidence of a causal relationship between DSMTs and liver failure. Age-stratified analyses demonstrated that liver failure-related deaths were concentrated among patients aged 55–74 years, with a notable increase in deaths in the 55–64 age group. This pattern may reflect a shift in competing risks: as advances in cancer treatment reduce deaths from rapid tumor progression, patients may survive long enough for liver-related comorbidities or complications, including treatment-related hepatotoxicity, liver metastases, or preexisting liver disease, to be more frequently recorded as contributing conditions (16, 17, 40). Racial and ethnic disparities were also evident. Although AAMRs declined across all racial and ethnic groups, the substantial increase in deaths among Hispanic patients suggests persistent structural disadvantages, potentially related to disparities in cancer screening, treatment access, socioeconomic status, and baseline liver disease burden (41, 42).

Geographic analyses further revealed pronounced regional heterogeneity in liver failure-related mortality among patients with DSMTs. Although declining trends were observed in both metropolitan and nonmetropolitan areas, differences in the magnitude and pace of decline suggest that variations in healthcare infrastructure, specialist availability, and access to advanced diagnostics and therapies may play an important role (43–45). Analyses by Census and HHS regions indicated that while some regions achieved substantial early improvements, progress slowed in later years, and mortality increased in certain regions. These findings emphasize the importance of incorporating liver function surveillance into region-specific chronic disease management frameworks for DSMTs and tailoring interventions to local population needs and healthcare capacities.

This study has several notable strengths. First, it was based on nationwide mortality data from CDC WONDER, encompassing all 50 U.S. states and the District of Columbia, thereby providing robust population representativeness and enabling assessment of temporal trends at a national scale. Second, by examining DSMTs as a pooled disease category, this study provides a broad population-level overview of deaths in which DSMTs and liver failure were co-recorded on death certificates. This approach allows identification of overall mortality surveillance patterns and disparities across demographic and geographic subgroups. In addition, the use of Joinpoint regression allowed identification of temporal inflection points, providing quantitative insight into the dynamic evolution of liver failure-related mortality and informing future public health surveillance, hypothesis generation, and more granular tumor site-specific research.

Several limitations should be acknowledged. First, death certificate data are subject to potential misclassification or underreporting of causes of death, particularly when multiple contributing conditions are present, and variation in reporting practices across regions and time periods may introduce information bias. Second, CDC WONDER lacks individual-level data on tumor stage, treatment modalities, liver metastasis status, baseline liver function, liver disease etiology, and underlying chronic liver disease, limiting mechanistic interpretation and precluding causal inference. Therefore, deaths in which DSMTs and liver failure were co-recorded should not be interpreted as deaths in which liver failure was necessarily caused by DSMTs. Third, DSMTs comprise heterogeneous tumor sites with distinct metastatic patterns, treatment landscapes, and relationships with underlying liver disease, and the inclusion of primary liver cancer is particularly important when interpreting pooled liver failure-related mortality estimates. As a result, the present analysis cannot distinguish whether liver failure-related mortality reflected primary hepatic malignancy, metastatic hepatic involvement, treatment-related hepatotoxicity, or preexisting liver disease. Finally, data suppression rules for small cell counts and the unavailability of certain stratified data in later years necessitated restrictions in some analyses, which may affect the precision and comparability of estimates.

In conclusion, this descriptive multiple cause-of-death surveillance study identified a substantial overall decline in deaths involving both DSMTs and liver failure among U.S. adults aged ≥45 years from 1999 to 2023. However, these gains were uneven across population subgroups and geographic regions, with recent trend deceleration or localized increases highlighting persistent vulnerabilities. These findings should be interpreted as pooled population-level mortality patterns rather than evidence of a shared etiologic mechanism across individual digestive tumor sites. Continued attention to liver function monitoring, underlying liver disease risk assessment, and targeted surveillance in high-risk populations may help reduce this mortality burden.

## Data Availability

The original contributions presented in the study are included in the article/**Supplementary Material**. Further inquiries can be directed to the corresponding authors.
